# Genetic Characterization of the Hemagglutinin Genes of Wild-Type Measles Virus Circulating in China, 1993–2009

**DOI:** 10.1371/journal.pone.0073374

**Published:** 2013-09-20

**Authors:** Songtao Xu, Yan Zhang, Zhen Zhu, Chunyu Liu, Naiying Mao, Yixin Ji, Huiling Wang, Xiaohong Jiang, Chongshan Li, Wei Tang, Daxing Feng, Changyin Wang, Lei Zheng, Yue Lei, Hua Ling, Chunfang Zhao, Yan Ma, Jilan He, Yan Wang, Ping Li, Ronghui Guan, Shujie Zhou, Jianhui Zhou, Shuang Wang, Hong Zhang, Huanying Zheng, Leng Liu, Hemuti Ma, Jing Guan, Peishan Lu, Yan Feng, Yanjun Zhang, Shunde Zhou, Ying Xiong, Zhuoma Ba, Hui Chen, Xiuhui Yang, Fang Bo, Yujie Ma, Yong Liang, Yake Lei, Suyi Gu, Wei Liu, Meng Chen, David Featherstone, Youngmee Jee, William J. Bellini, Paul A. Rota, Wenbo Xu

**Affiliations:** 1 Regional Reference Measles Laboratory for the WHO Western Pacific Region, Key Laboratory of Medical Virology Ministry of Health, National Institute for Viral Disease Control and Prevention, China Center for Disease Control and Prevention, Beijing, China; 2 Division of Viral Diseases, Centers for Disease Control and Prevention, Atlanta, Georgia, United States of America; 3 Shanghai Center for Disease Control and Prevention, Shanghai City, China; 4 Henan Center for Disease Control and Prevention, Zhengzhou City, Henan Province, China; 5 Shandong Center for Disease Control and Prevention, Jinan City, Shandong Province, China; 6 Shanxi Center for Disease Control and Prevention, Taiyuan City, Shanxi Province, China; 7 Tianjin Center for Disease Control and Prevention, Tianjin City, China; 8 Chongqing Center for Disease Control and Prevention, Chongqing City, China; 9 Hainan Center for Disease Control and Prevention, Haikou City, Hainan Province, China; 10 Sichuan Center for Disease Control and Prevention, Chengdu City, Sichuan Province, China; 11 Liaoning Center for Disease Control and Prevention, Shenyang City, Liaoning Province, China; 12 Shaanxi Center for Disease Control and Prevention, Xian City, Shannxi Province, China; 13 Anhui Center for Disease Control and Prevention, Hefei City, Anhui Province, China; 14 Jilin Center for Disease Control and Prevention, Changchun City, Jilin Province, China; 15 Hunan Center for Disease Control and Prevention, Changsha City, Hunan Province, China; 16 Guangdong Center for Disease Control and Prevention, Guangzhou City, Guangzhou Province, China; 17 Xinjiang Center for Disease Control and Prevention, Urumchi City, Xinjiang Province, China; 18 Jiangsu Center for Disease Control and Prevention, Nanjing City, Jiangsu Province, China; 19 Zhejiang Center for Disease Control and Prevention, Hangzhou City, Zhejiang Province, China; 20 Jiangxi Center for Disease Control and Prevention, Nanchang City, Jiangxi Province, China; 21 Qinghai Center for Disease Control and Prevention, Xining City, Qinghai Province, China; 22 Ningxia Center for Disease Control and Prevention, Yinchuan City, Ningxia Province, China; 23 Fujian Center for Disease Control and Prevention, Fuzhou City, Fujian Province, China; 24 Heilongjiang Center for Disease Control and Prevention, Harbin City, Heilongjiang Province, China; 25 Hebei Center for Disease Control and Prevention, Shijiazhuang City, Hebei Province, China; 26 Hubei Center for Disease Control and Prevention, Wuhan City, Hubei Province, China; 27 Inner Mongolia Center for Disease Control and Prevention, Hohhot City, Inner Mongolia Province, China; 28 Guangxi Center for Disease Control and Prevention, Nanning City, Guangxi Province, China; 29 Beijing Center for Disease Control and Prevention, Beijing City, China; 30 Immunization, Vaccines and Biologicals, World Health Organization, Geneva, Switzerland; 31 Expanded Programme on Immunization, Western Pacific Regional Office, World Health Organization, Manila, Philippines; Naval Research Laboratory, United States of America

## Abstract

**Background:**

China experienced several large measles outbreaks in the past two decades, and a series of enhanced control measures were implemented to achieve the goal of measles elimination. Molecular epidemiologic surveillance of wild-type measles viruses (MeV) provides valuable information about the viral transmission patterns. Since 1993, virologic surveillnace has confirmed that a single endemic genotype H1 viruses have been predominantly circulating in China. A component of molecular surveillance is to monitor the genetic characteristics of the hemagglutinin (H) gene of MeV, the major target for virus neutralizing antibodies.

**Principal Findings:**

Analysis of the sequences of the complete H gene from 56 representative wild-type MeV strains circulating in China during 1993–2009 showed that the H gene sequences were clustered into 2 groups, cluster 1 and cluster 2. Cluster1 strains were the most frequently detected cluster and had a widespread distribution in China after 2000. The predicted amino acid sequences of the H protein were relatively conserved at most of the functionally significant amino acid positions. However, most of the genotype H1 cluster1 viruses had an amino acid substitution (Ser240Asn), which removed a predicted N-linked glycosylation site. In addition, the substitution of Pro397Leu in the hemagglutinin noose epitope (HNE) was identified in 23 of 56 strains. The evolutionary rate of the H gene of the genotype H1 viruses was estimated to be approximately 0.76×10^−3^ substitutions per site per year, and the ratio of *dN* to *dS* (*dN*/*dS*) was <1 indicating the absence of selective pressure.

**Conclusions:**

Although H genes of the genotype H1 strains were conserved and not subjected to selective pressure, several amino acid substitutions were observed in functionally important positions. Therefore the antigenic and genetic properties of H genes of wild-type MeVs should be monitored as part of routine molecular surveillance for measles in China.

## Introduction

Measles virus (MeV), a member of the genus *Morbillivirus* of the family *Paramyxoviridae*, is an enveloped virus with a nonsegmented negative-sense RNA genome. Measles virus is highly contagious and causes a disease characterized by high fever, cough, coryza, conjunctivitis, and appearance of a maculopapular rash [1]. Despite the existence of an effective vaccine, many developing countries are still experiencing endemic measles. In China, measles remains a major public health concern because of frequent outbreaks although the two effective vaccines, Shanghai191 (S191) and Changchun47 (C47) have been widely used to prevent illness. In 2005, China set a goal of measles elimination by 2012, and the goverment has been making great efforts to achieve this goal including strengthening routine immunization, expanding epidemiologic and virologic surveillance, increasing vaccination of internal mobile poopulations, and initiation of large-scale supplemental immunization acitivities (SIA). For example, the SIA of September 2010 targeted 100 million children and teenagers.

MeV is a monotypic virus, but 24 genotypes (A, B1–B3, C1–C2, D1–D11, E, F, G1–G3 and H1–H2) have been described [2]–[4]. In China, genotyping of wild-type MeV strains circulating during 1993–1994 led to identification of a novel genotype, H1 [5]. Since then, continuous molecular surveillance revealed that genotype H1 MeVs were endemic throughout mainland China. The genotype H1 sequences could be divided into 2 clusters based on phylogenetic analysis of the 450 nucleotides coding for the carboxyl-terminus of the nucleoprotein (N) gene (N-450). Cluster1 viruses have been the most frequently detected strains since 2000 and cluster2 viruses were not detected after 2005 [6]–[10].

The genome of MeV contains six genes that encode the nucleoprotein (N), phosphoprotein (P), matrix (M), fusion (F), hemagglutinin (H), and polymerase (L) proteins. The H protein, an 80 kilodalton (kD) glycoprotein, is responsible for receptor-binding and is the major target for neutralizing antibodies [11]. The H protein usually contains five potential N-linked glycosylation sites which are clustered at amino acid positions 168, 187, 200, 215, 238. Some genotypes have an additional N-linked glycosylation site at position 416 [12], [13]. MeV uses up to three cellular receptors, including Signaling Lymphocyte Activation Molecule (SLAM, CD150), CD46 and nectin-4 [14], [15], [16], [17] . The H protein contains 13 cysteine residues, seven of which are located at amino acid positions 287, 300, 381, 394, 494, 579, 583. These amino acids play a critical role in maintaining the antigenic structure of the H protein [18]. A few linear neutralizing epitopes have been identified using monoclonal antibodies [19], [20].

We carried out this study to describe the genetic variability of the H genes of MeVs circulating throughout China over a 17-year period. This work provides the first detailed report of the sequences of the H genes of Chinese MeVs as well as a unique opportunity to analyze genetic changes in the H genes from a single genotype of MeV over an extended period of time.

## Results

### The epidemiologic profile of measles in China

**Figure 1:**
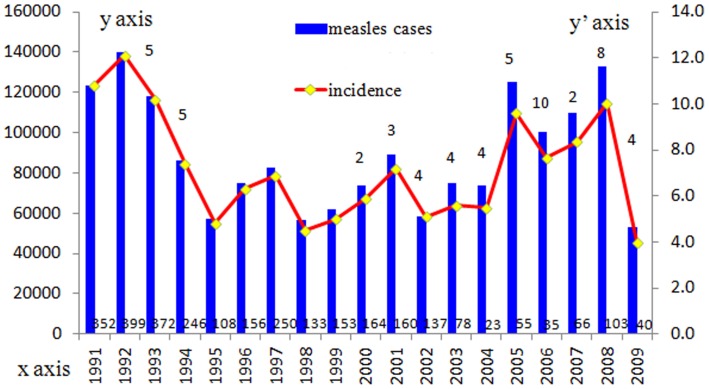
Reported measles cases and incidence in China, 1991–2009. The number above the column represents the number of representative measles strains selected for the complete H gene sequence analysis. Blue bars indicate the number of reported measles cases and yellow solid diamonds indicate the incidence (/100,000 population) of each year, the Arabic numerals above the x-axis indicates the number of deaths. X-axis denotes year, y-axis on left denotes reported number of cases and y-axis on right denotes the incidence per 100,000 population.

The number of reported measles cases and deaths and the incidence of measles were obtained from the National Notifiable Disease Report System (NNDRS) (Figure 1). Nationwide outbreaks of measles occurred every 3–4 years because of the accumulation of susceptible children, especially in areas with lower routine immunization coverage. Viral isolates were obtained throughout the period shown in Figure 1.

### Genetic characterization of the H genes of Chinese MeVs

**Figure 2:**
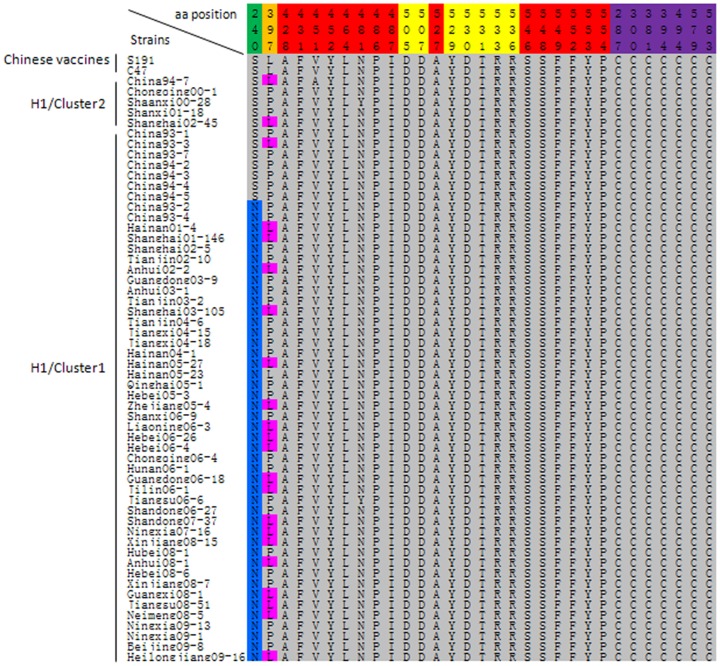
Alignment of the predicted amino acid sequences of the partial H gene. The variation of amino acid Ser240Asn (highlighted in blue) leads to the absence of a glycosylation site; the exchange of Pro397Leu (highlighted in pink) that results in loss of recognition of two monoclonal antibodies directed against HNE; the amino acid residues of putative binding sites for CD46, SLAM and seven cysteine residues were highlighted in red, yellow and purple, respectively.

The H glycoprotein is the major target for neutralizing antibodies directed against the virus. For this report, 46 new H gene sequences were obtained from viral isolates and compared to 10 previously published H gene sequences. Analysis of the predicted amino acid sequences of the H proteins showed that genotype H1 cluster1 isolates circulating in 2000–2009 had a conserved substitution of Ser240Asn, which removed the predicted N-linked glycosylation site at amino acid 238 (Figure 2). In addition, 23 of 56 genotype H1 strains showed an exchange of Pro397Leu, this 397 amino acid is a part of the linear hemagglutinin noose epitope (HNE) which is located at amino acids 379–410. However, all of the putative binding sites for SLAM and CD46 were conserved (Figure 2). The seven cysteine residues presumably responsible for the antigenic structure of the H molecules (amino acids 287, 300, 381, 394, 494, 579, and 583) were highly conserved [18]. The amino acids 236–250 predicting B Cell epitopes (BCE) were relatively conserved [21], excluding the previously described substitutions at amino acids 240 and 243 in the Chinese vaccine strains (Arg243, Gly243).

**Figure 3:**
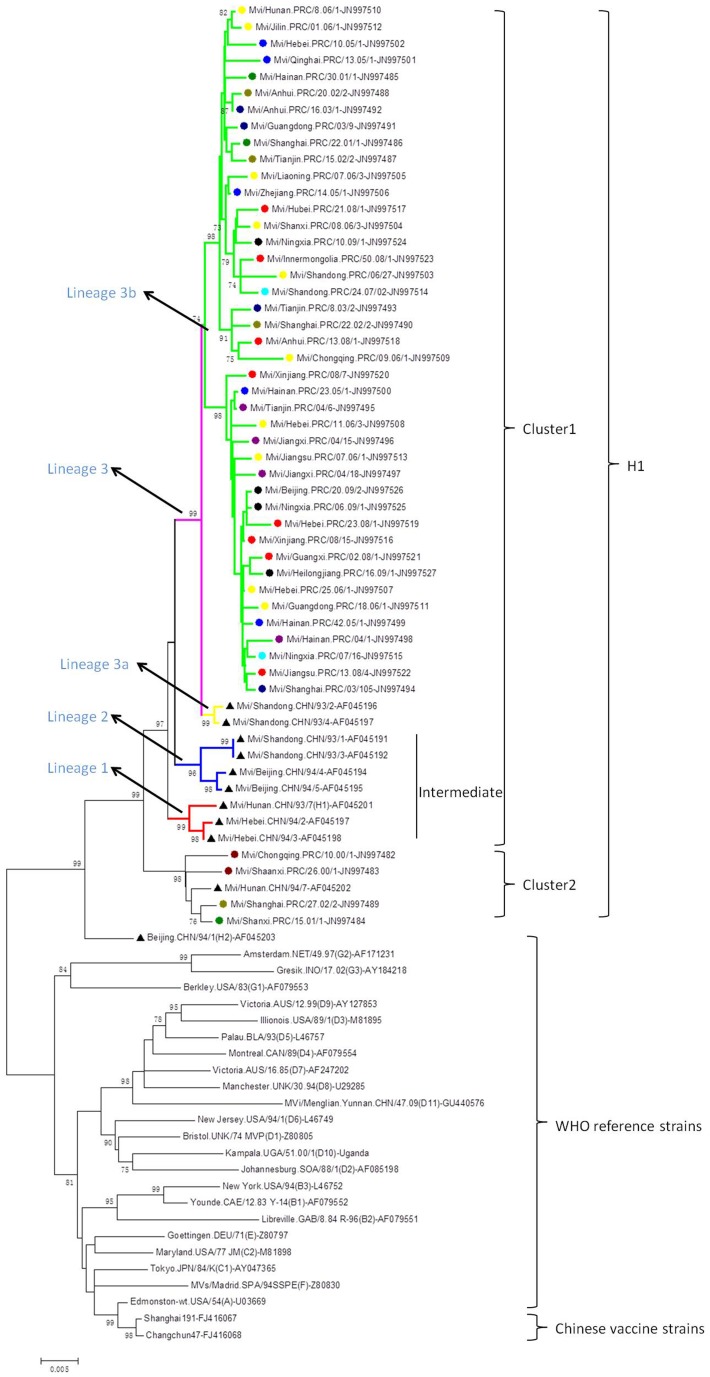
Phylogenetic relationship based on the complete H gene sequences. Neighbour-joining tree was reconstructed with full-length H gene sequences from 56 genotype H1 wild-type measles isolates from mainland China, the WHO reference strains of each genotype and Chinese vaccine strains. The sequences of the circulating strains in 1993–1994 and in 2000–2009 are indicated by symbol “▴” and “•”, respectively, each color of symbol “•” represents the annually circulating strains. The genotype H2 reference strain below cluster 2 which is also marked by a triangle was identified in 1994, China. The branches for the different lineages are marked by various colors. The WHO standard name of MeVs and GenBank accession numbers of all the sequences are available in the figure. Numbers at nodes represent the percentage of 1,000 bootstrap replicates (values <70 are not shown). Bar, 0.005 nucleotide substitutions per site.

The complete H gene sequences of 56 H1 MeVs, the Chinese vaccine strains, and other genotype reference strains were used for phylogenetic analysis (Figure 3). The sequences of genotype H1 isolates could be divided into at least 2 clusters without obvious chronological and geographical distributions. Fifty-one isolates belonged to cluster1 which also contained a few more diverse strains isolated in 1993–1994. Five isolates belonged to cluster2. The topology of the tree suggested that multiple chains of virus transmission were present and the sequences showed no temporal or geographic distribution (Figure 3).

The evolutionary rates of the H gene for the genotype H1 and H1 cluster1 viruses were 0.76×10^−3^ and 0.65×10^−3^ substitutions per site per year, respectively. The *dN* and *dS* were calculated for selection pressure analysis, the results indicated the *dN/dS* ratios for the genotype H1 and H1 cluster1 were 0.21 and 0.20, respectively.

## Discussion

The results of the sequence analysis reported here provide another example of the genetic stability of MeV. The H genes of 56 MeVs isolated over a 17 year time span showed remarkable conservation of functionally important amino acids. In particular, the stability of the amino acids comprising the receptor-binding sites likely contributes to the monotypic nature of MeV [22]–[24]. The cysteine residues responsible for the tertiary structure of the H molecule were also highly conserved [18] . The amino acid substitutions detected appeared to be the result of a gradual accumulation of genetic changes and the degree to which these amino acid mutations contribute to antigenic changes of the H protein is being investigated.

The H protein of MeV usually contains five or six potential N-linked glycosylation sites. The fifth site, located at amino acid 238, was absent in all the H1 cluster1 strains circulating in 2000–2009. Hu *et al.* showed that the fifth glycosylation site had minimal impacted on the processing and antigenicity of the H molecule, though the other glycosylation sites played important functional roles [25], [26]. Shi *et al.* demonstrated that the neutralization titers of serum samples from human vaccinees were lower against some of the wild-type viruses than against vaccines strains and antigenic differences have been detected by monoclonal antibodies directed against the H protein [27], [28]. The linear hemagglutinin noose epitope (HNE; amino acids 379–410) was highly stable in both vaccine and wild-type strains [29]–[31]. We found that nearly half of the genotype H1 strains showed an exchange of Pro397Leu, a mutation that results in loss of recognition of two monoclonal antibodies directed against HNE [32]. Though the H protein of a few genotype H1 MeVs would presumably not be recognized by antibodies directed against the HNE, serum from human vaccines neutralized H1 MeVs with either Pro or Leu at position 397 [36]. This conservation of neutralizing epitopes was also noted by Bouche *et al.* [33]. The strong conservation of the seven cysteine residues also clarified the antigenic structure and processing of the H molecules [18]. The Chinese vaccines have been very effective at dramatically reducing the incidence of measles in China. However, since amino acid substitutions were observed on the H protein between genotype H1 strains and the Chinese vaccine strains, it will be important to continually monitor the antigenicity and genetic characteristics of the H protein of circulating MeVs.

Molecular surveillance of wild-type MeVs from 1993 to 2008 based on analysis of the 450 nucleotides coding for the carboxyl-terminus of the N gene indicated that genotype H1 cluster1 viruses were continuously circulating in China [6], [9], [10]. The phylogenetic tree constructed based on the H gene sequences, confirmed the analysis based on the N-450 sequences that genotype H1 cluster1 viruses were the predominant strains in China during 1993–2009. Cluster1 was divided into 3 lineages and lineages 1 and 2 were considered to be an intermediate group that appeared to be the predominant strains in 1993–1994 and have not been found since 1995 [6]–[8]. Lineage 3 was further divided into lineage 3a and 3b and all of the strains isolated from 2000–2009 belonged to lineage 3b. Genotype H1 cluster2 viruses have not been detected since 2002. Although there was a temporal distribution among the MeV strains of the 3 lineages of cluster1, multiple transmission chains of were apparent in lineage 3b without an obvious temporal distribution (Figure 3). The observation that the phylogenetic analysis based on the H gene sequences gave results that were consistent with results obtained after analysis of the much larger set of N-450 sequences suggests that the H gene sequences provide a reasonable representation of MeVs circulating in China. The caveat is that virologic surveillance is incomplete and we cannot be sure that all of the lineages were represented by the H gene or N-450 data sets.

Viral RNA polymerases have high error rates due to the lack of proofreading ability, which leads to the high mutation rate of the viral genome and rapid evolutionary rates. In some cases, these mutations allow the virus to adapt to a new host or escape the host's anti-viral responses. This study estimated that the mutation rate of the predominant group (Cluster1) of genotype H1 MeVs was 0.65×10^−3^ substitutions per site per year based on the analysis of the complete H genes. This rate is slightly higher than the rates previously reported by Jenkins *et al*. (0.4×10^−3^) and Rima *et al*. (0.5×10^−3^), but it is low when compares with some other RNA viruses such as enterovirus 71 (3.18×10^−3^), human influenza A (1.8×10^−3^), and equivalent to the mutation rates reported for dengue virus and human rotavirus [34]–[36]. The *dN* to *dS* ratios for both H1 and H1 cluster1 were <1 that demonstrating that the H gene of the MeVs analyzed was not subject to antigenic selection. Rather, the data suggest that the amino acid substitutions in the H gene were the result of random genetic drift, rather than accumulated mutations.

In summary, the H gene of the MeVs endemic in China should be monitored continuously for genetic variations that could affect antigenic properties. Our group is currently using the bioinformatics methods to understand the disappearance of cluster2 and map the antigenic domains on the 3-dimentional crystal structure of the H protein. This information will be helpful to evaluate the efficiency of the current vaccines.

## Materials and Methods

### Epidemiologic information

As a class B reportable disease, suspected measles cases are reported to the National Notifiable Disease Reporting System (NNDRS) in China. The number of measles cases, the annual measles incidence, and measles-associated deaths were retrieved directly from NNDRS reports.

### Selection and isolation of viruses

In 2001, the National Measles Laboratory Network was established to carry out measles surveillance in China. Genotyping and genetic analysis of wild-type viruses were included in the laboratory surveillance. These strains were chosen from viral strains bank of the National Measles Laboratory (NML) to achieve representative chronological and geographical distributions (covering 25 provinces). All of the selected strains were cultured and propagated in Vero/hSLAM cells, which were maintained in minimal essential medium supplemented with 2% fetal bovine serum [37]. Viruses were harvested when the classic measles CPE was maximal.

### Determination of the complete H gene nucleotide sequences

Total viral RNA was extracted from the infected Vero/hSLAM cells with the QIAamp Viral RNA mini kit (Qiagen, Valencia, CA) according to the manufacturer's instruction. The entire protein-coding region of the H gene was amplified with the sequence-specific primers (Table 1) by using the SuperScript™III One-Step RT-PCR kit (Invitrogen, Carlsbad, CA). The PCR products were sequenced directly after purification (QIA gel extraction kit, Qiagen, Valencia, CA) by the dye terminator method (BigDye Terminator, version 3.1, cycle sequencing kit; Applied Biosystems) in an ABI PRISM 3100 genetic analyzer (Applied Biosystems, Hitachi, Japan). The primers used for sequencing are listed in Table 1.

**Table 1:** Primers used for amplification and sequencing of the entire H gene.

Primer^a^	Sequence(5′-3′orientation)	Positions^b^	Amplicon^c^
MHs	GTGCAAGATCATCCACAATGTCACC	7254-7278	1,919 bp
MHas	GTATGCCTGATGTCTGGGTGA	9172-9152	
H1s	GTGCAAGATCATCCACAATGTCACC	7254-7278	Seq
H2as	GTCAGAGATGAATTTCAC	7630-7613	Seq
H3s	TTGGTGAACTCAACTCTACTG	7766-7786	Seq
H4as	GGA ACTGAGTTTGACATCAC	7811-7792	Seq
H5as	GTATGCCTGATGTCTGGGTGA	9172-9152	Seq

a : s, sense orientation; as, antisense orientation.

b : the primers' nucleotide locations and range on the basis of the Measles prototype strain: Edmonston complete genome (GenBank ID: AF266288).

c : Length of the PCR product or use of the primer for sequencing (Seq).

### Sequence and phylogenetic analysis

Sequence data were assembled and edited using Sequencher software version 4.0.5 (GeneCodes, Ann Arbor, MI); sequence alignments were assembled with BioEdit version 7.0 (Tom Hall, North Carolina State University, Raleigh, NC), and phylogenetic trees were constructed by the neighbor-joining method (1000 bootstraps) using MEGA version 4.0 [38], [39]. The 24 WHO reference genotype strains, 56 genotype H1 strains and two Chinese vaccine strains were used to construct the phylogenetic trees.

### Estimation of evolutionary rate

Because of the lack of a true “ancestor” strain and the apparent presence of multiple lineages, to estimate the evolutionary rate of genotype H1 and genotype H1 cluster1 viruses, we used the oldest strain in each target group as a reference. The evolutionary distances were calculated by overall mean scope according to the Kimura two-parameter method of the MEGA program. The *dS* (synonymous substitutions per synonymous site), *dN* (non-synonymous substitutions per non-synonymous site), and the ratios of *dS* to *dN* values also were analyzed.

### Nucleotide sequence accession numbers

Nucleotide sequences of the complete H gene (1854 nucleotides) of the 46 measles virus strains which were determined have been submitted and deposited in the GenBank database, the accession numbers are JN997482 to JN997527. Accession numbers of Chinese circulating strains in 1993–1994, all the WHO reference strains of each genotype and Chinese vaccine strains are given in Figure 3.
